# Targeted Nerve Implantation: Intramuscular Coaptation in a Denervated Motor Endplate Zone for Below and Above the Knee Amputations

**DOI:** 10.7759/cureus.115619

**Published:** 2026-09-01

**Authors:** Dev Patel, Diana Mastellone, Jessieka Knazze, Steven Bonawitz, Michael J Franco

**Affiliations:** 1 Orthopaedics, Cooper Medical School of Rowan University, Camden, USA; 2 Medical School, Cooper Medical School of Rowan University, Camden, USA; 3 Plastic and Reconstructive Surgery, Cooper University Health Care, camden, USA; 4 Plastic and Reconstructive Surgery, Cooper University Health Care, Camden, USA; 5 Surgery, Division of Plastic and Reconstructive Surgery, Cooper University Hospital, Camden, USA

**Keywords:** lower limb amputation, neuroma phantom limb pain, peripheral nerve surgery, post-amputation pain, regenerative peripheral nerve interface, targeted muscle reinnervation, targeted nerve implantation

## Abstract

Post-amputation neuropathic pain, including symptomatic neuroma pain and phantom limb pain (PLP), remains a significant source of morbidity following lower limb amputation and can substantially impair prosthetic use and quality of life. Targeted muscle reinnervation (TMR) has demonstrated effectiveness in reducing these complications by providing transected nerves with physiologic motor targets; however, its application may be limited by recipient motor nerve caliber mismatch, inadequate target availability, or unfavorable anatomy. Regenerative peripheral nerve interface (RPNI) offers an alternative but requires free muscle graft harvest and is associated with increased operative complexity. Targeted nerve implantation (TNI) is a hybrid technique that implants a transected donor nerve directly into the zone of relative denervation near a native motor entry point without requiring motor nerve coaptation or free muscle grafting. We describe our surgical technique for TNI in above-knee and below-knee amputations, highlighting technical considerations and indications when standard TMR is not feasible. By utilizing denervated local muscle as a biologic target for axonal ingrowth, TNI preserves the physiologic principles of nerve regeneration while avoiding the limitations of both TMR and RPNI. TNI represents a practical adjunct for managing anatomical constraints encountered during amputation surgery. Further prospective studies are needed to evaluate postoperative pain, neuroma formation, phantom limb pain, prosthetic tolerance, and patient-reported functional outcomes.

## Introduction

Limb amputations are one of the earliest documented surgical procedures, with archeological evidence dating from over 31,000 years ago in Borneo [1]. While early amputations were likely rudimentary, modern techniques have evolved significantly, incorporating advanced surgical methods and prosthetic technologies. In the modern day, 150,000 lower limb amputations (LLA) occur yearly in the United States. Diabetes-related complications, peripheral vascular disease, infection, and trauma are the leading causes of lower limb amputation [2]. Despite significant advancements in surgical techniques and prosthetic fitting, patients continue to experience substantial morbidity following amputation, with post-amputation pain remaining a major contributor to impaired prosthetic use and reduced quality of life [3]. 

A key modifiable factor affecting quality of life post-amputation is residual nerve pain, which may be due to neuroma formation or phantom limb pain (PLP). Neuromas are benign proliferations of neural tissue that develop when a nerve is severed and left without a distal muscle target [4]. Up to 15% of amputees may develop neuromas [4]. The significant pain associated with neuromas may limit or completely prevent prosthetic use. Local irritation due to neuromas also makes prosthetic fitting a challenge, as care must be taken to distribute force in areas not affected by the neuroma [3]. In addition to neuroma-related pain, up to 80% of patients post-amputation suffer from PLP [5]. PLP is a complex neuropathic pain condition hypothesized to involve dysfunction of the severed peripheral nerves at the amputation site and erroneous relay to the central nervous system [6]. These post-amputation complications have massive impacts on amputees. Recent data have shown that PLP is significantly associated with increased odds of major depressive disorder, generalized anxiety disorder, posttraumatic stress disorder, and suicidal ideation [7]. Pharmacological therapies have demonstrated limited efficacy for post-amputation pain, and rather surgical techniques, including targeted muscle reinnervation (TMR) and regenerative peripheral nerve interfaces (RPNI), have shown more promise in controlling pain [8].

TMR is an innovative technique used to aid in the sequelae of post-amputation neuropathic pain. The technique involves redirecting transected peripheral sensory nerves to residual target muscles. TMR facilitates physiological nerve regeneration into the motor endplate zone and has been associated with reductions in both neuroma formation and phantom pain [9]. In a systematic review (n=448 limbs that underwent TMR), all studies reviewed showed a reduction in PLP and residual limb pain (RLP) described as discomfort in the remaining extremity post-amputation following TMR [10]. However, TMR may not always be feasible due to lack of suitable recipient motor nerves and donor-recipient caliber mismatch leading to TMR failure. In a case series, donor nerve redundancy, kinking, size mismatch, superficial placement of the nerve coaptation, poor target selection, and incomplete target muscle denervation were identified as factors that may lead to TMR failure [11]. When suitable motor nerve targets are unavailable, alternative techniques such as RPNI have been utilized. RPNI involves implanting transected nerves into free muscle grafts, thereby providing a denervated biologic target for axonal ingrowth [12]. This technique, however, has its own limitations, including longer operative times, graft donor site complications, and risk of graft failure [13]. Targeted nerve implantation (TNI) is a hybrid between traditional TMR and RPNI, combining the use of local muscle targets without reliance on motor nerve coaptation or free muscle grafting.

TNI was first described by Pet et al. in a 2014 case series of patients undergoing TNI. The results were promising, with 11 of 12 patients (92%) after primary TNI and 20 of 23 patients (87%) after secondary TNI pain-free [14]. From a search of the literature on PubMed, in the 12 years since that study was performed, there have been no additional studies discussing their experience with TNI, and rather just systematic reviews that reference Pet’s results. This article aims to build upon Pet’s description of how TNI is performed, to describe the operative approach and indications for TNI, and its utility in above-the-knee and below-the-knee amputations for neuropathic pain reduction.

## Technical report

Standard TMR technique

For below-knee amputation (BKA) standard TMR in the setting of a BKA, we utilize a posterior approach through the popliteal fossa. A zigzag or curvilinear incision is made, and the tibial and common peroneal nerves are identified and exposed. At this level, we identify motor branches, most commonly to the gastrocnemius. In our experience, it is more challenging to reliably identify pure motor branches to the soleus distally. We follow the general principle of donor distal and recipient proximal when selecting targets. The sural nerve is identified as it receives contributions from both the tibial and common peroneal nerves. Unless it has been directly injured, it is preserved to maintain sensibility of the posterior flap. In most cases, there is a reasonable size match between the tibial or common peroneal nerves and the gastrocnemius motor branches. These motor branches can be further dissected into smaller fascicular groups if needed to improve the match. 

For above-knee amputation (AKA), a similar posterior approach is used. The sciatic nerve is identified and dissected into its tibial and peroneal components. Motor branches to the posterior thigh musculature are then identified, again following the principle of donor distal and recipient proximal. Standard coaptation is performed when size match is adequate. Size mismatch is more common in AKA cases.

The recipient motor branch is dissected to a length sufficient to permit tension-free coaptation to the donor nerve; no fixed length is specified, as this varies with amputation level and available branch anatomy. A nerve stimulator can be helpful for confirming motor branches intraoperatively but is not considered necessary. Once an adequate target is confirmed, standard end-to-end epineural coaptation is performed using 6-0 Prolene, with the coaptation site kept tension-free through the expected range of motion of the residual limb; this is prioritized over precise nerve length or exact target selection.

When a size mismatch is present and the recipient motor branch is too small, we transition to TNI, described below Figure 1, original image created in Procreate (Savage Interactive, Hobart, Australia). If this is not feasible, an RPNI technique may be considered.

**Figure 1:**
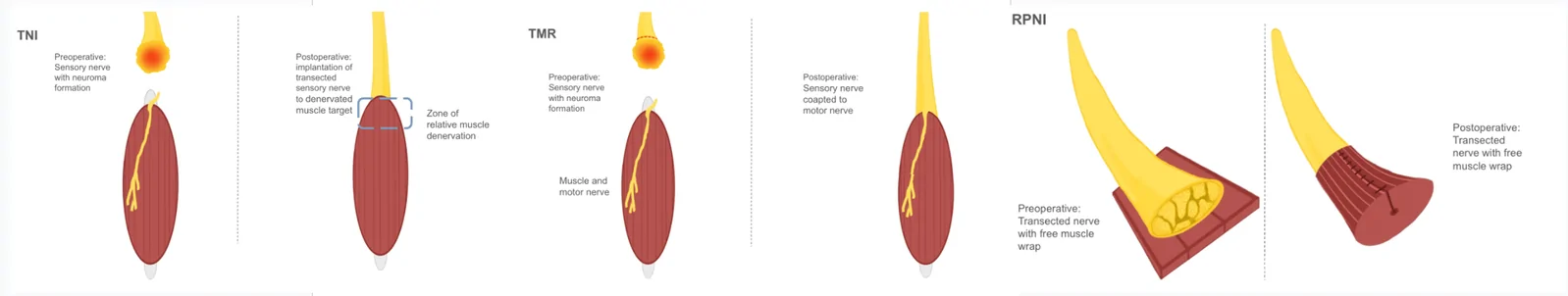
Pictorial Representation of TNI, TMR and RPNI Original image created in Procreate (Savage Interactive, Hobart, Australia) by Christina Mastellone.

TNI technical approach

For TNI in the zone of relative denervation, we follow the general exposure used in standard targeted muscle reinnervation (TMR). The donor nerve is dissected distally, and the recipient motor branch is identified proximally to confirm motor function and appropriate target selection. A nerve stimulator is a useful adjunct for identifying and confirming motor branches and for defining the motor entry region when anatomy permits, but it is not necessary in every case.

When there is a size mismatch, the recipient motor branch is traced distally to its point of entry into the muscle. This location may be marked with a suture to define the intended zone of relative denervation. The zone of denervation refers to a region of muscle or tissue that has lost its nerve supply, typically due to injury, disease, or surgical transection of the supplying nerve. In this zone, muscle fibers lack active neural input and are therefore susceptible to atrophy and fibrosis over time. In the context of nerve reconstruction and TMR, identifying and targeting denervated muscle regions is essential for successful reinnervation, as these areas provide available motor end plates to receive regenerating axons from the donor nerve.

The recipient motor branch is then sharply divided at the nerve-muscle interface. Rather than performing a traditional end-to-end coaptation, the donor nerve is inset directly into the muscle at this marked entry zone. Sufficient donor nerve is mobilized to permit healthy proximal transection and completely tension-free placement into the selected muscle target; no fixed length is specified, as this varies considerably with anatomy and amputation level. The target muscle is chosen for adequate health, vascularity, and volume to fully surround and protect the implanted nerve, favoring the region around the native motor entry point when feasible. When the donor nerve harbors a symptomatic neuroma, it is resected proximally until healthy-appearing fascicles are encountered before implantation.

A small surrounding muscle cuff (approximately 1 cm) is secured to the epineurium of the donor nerve using interrupted 6-0 Prolene sutures, achieving secure fixation without strangulation or compression of the nerve. An intramuscular pocket is created at or near the selected motor entry region, and the donor nerve is inset deeply enough to be surrounded by vascularized muscle rather than lying superficially against fascia or scar. The goal is to position the donor nerve within a region of relatively denervated muscle near the native motor entry point, where motor endplate density is expected to be highest, to facilitate axonal ingrowth. The nerve must lie naturally within the muscle without traction through the full expected range of motion and without a vector tending to pull it out of the muscle - perhaps the most critical reproducible technical point in this technique. Adequate intramuscular depth, circumferential muscle coverage, secure epineurium-to-muscle fixation, and elimination of tension are the primary measures used to prevent migration.

Exact nerve length, implantation depth, and muscle-cuff dimensions have not been experimentally optimized and represent areas for future investigation. Figures 2-4 demonstrate the variability in TNI approach, reflecting modifications necessitated by amputation level and patient-specific anatomical and clinical considerations.

**Figure 2:**
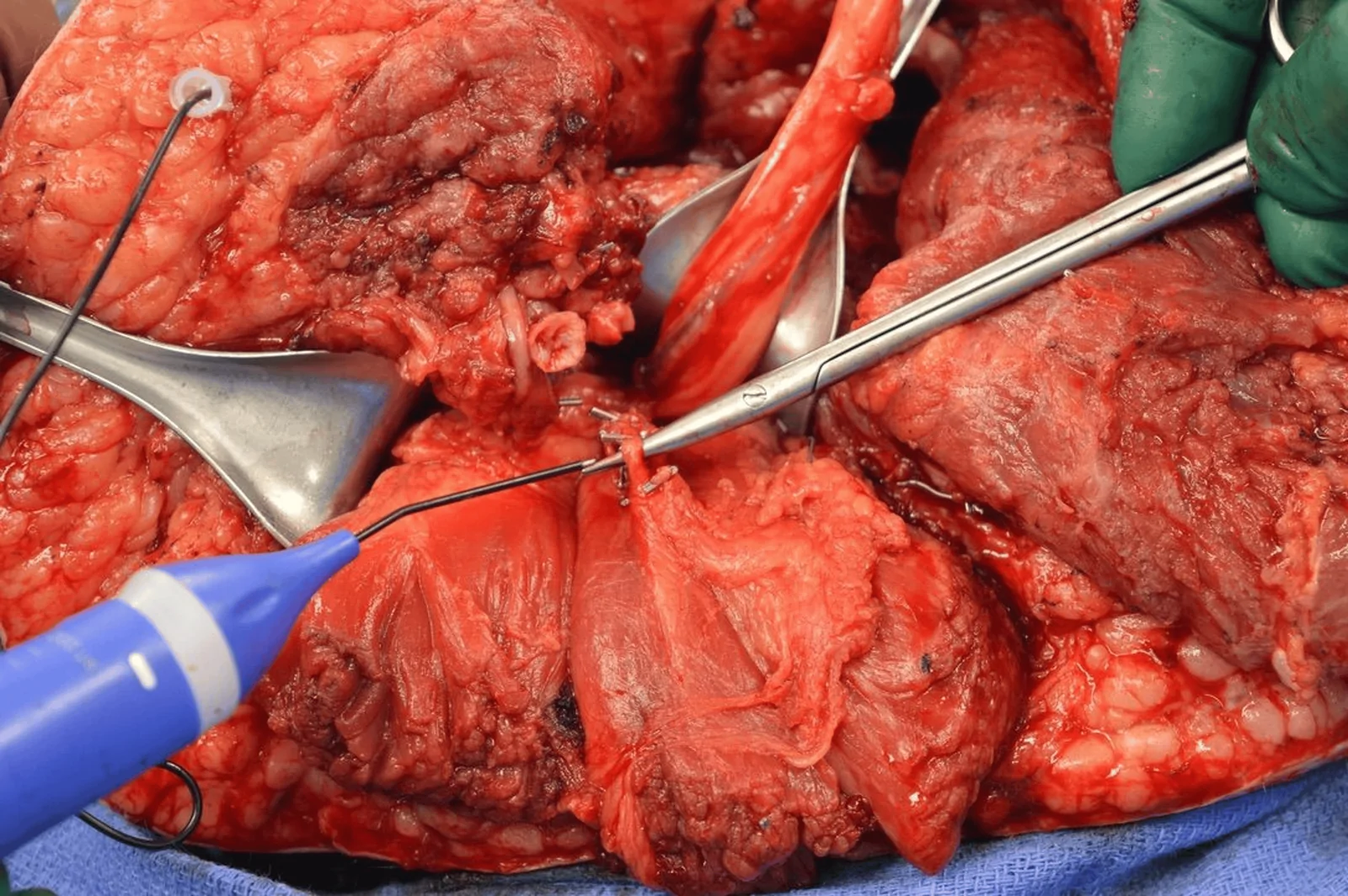
Intraoperative view demonstrating dissection of the sciatic nerve and identification of a recipient motor branch for targeted nerve intervention (TNI) in the zone of relative denervation.

**Figure 3:**
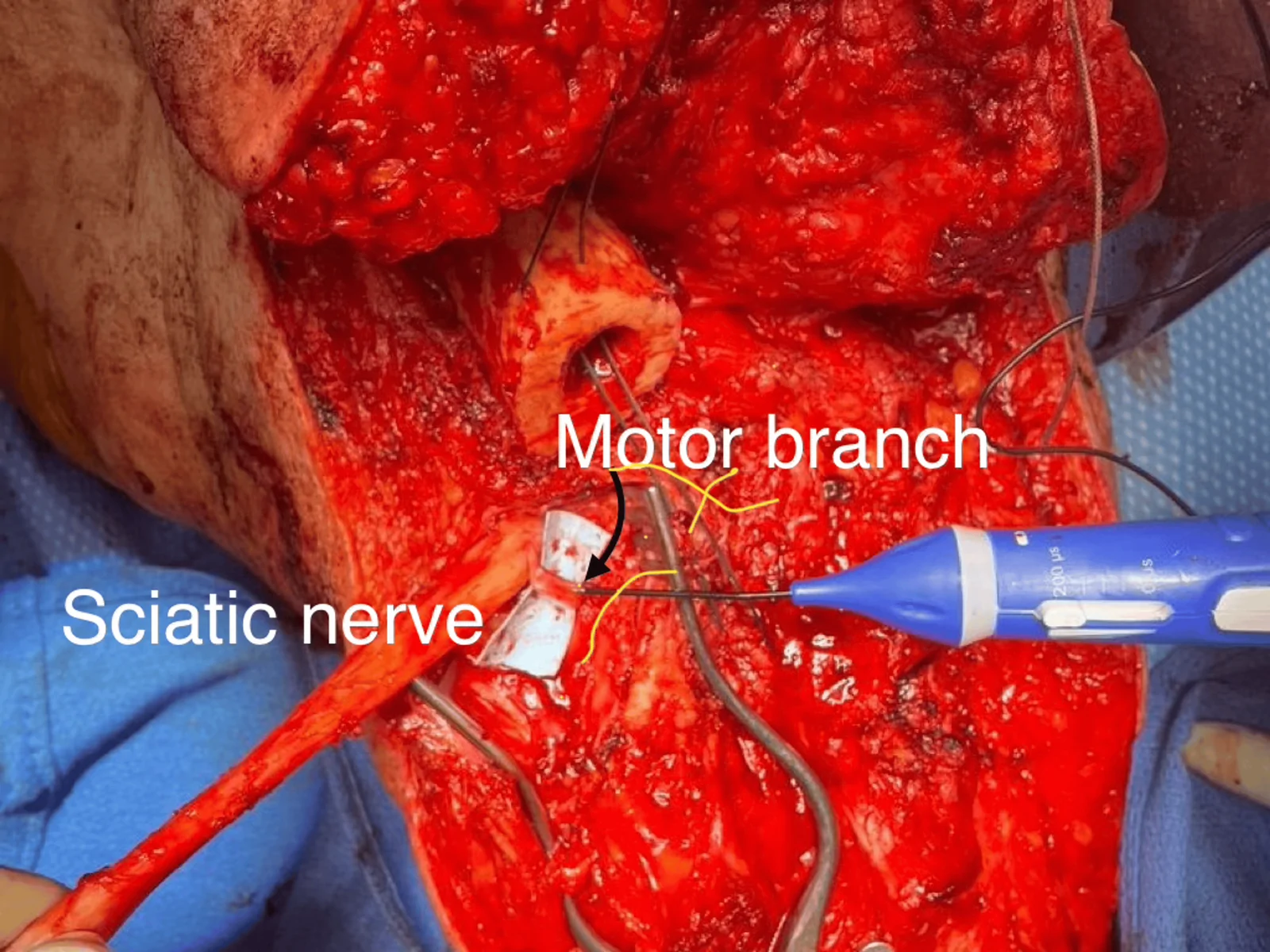
Intraoperative view demonstrating a variation of targeted nerve implantation (TNI) performed in an above-knee amputation. The procedure presented notable challenges secondary to limited residual nerve length and the need to accommodate nerve routing through the stump, further complicated by the inability to achieve prone positioning.

**Figure 4:**
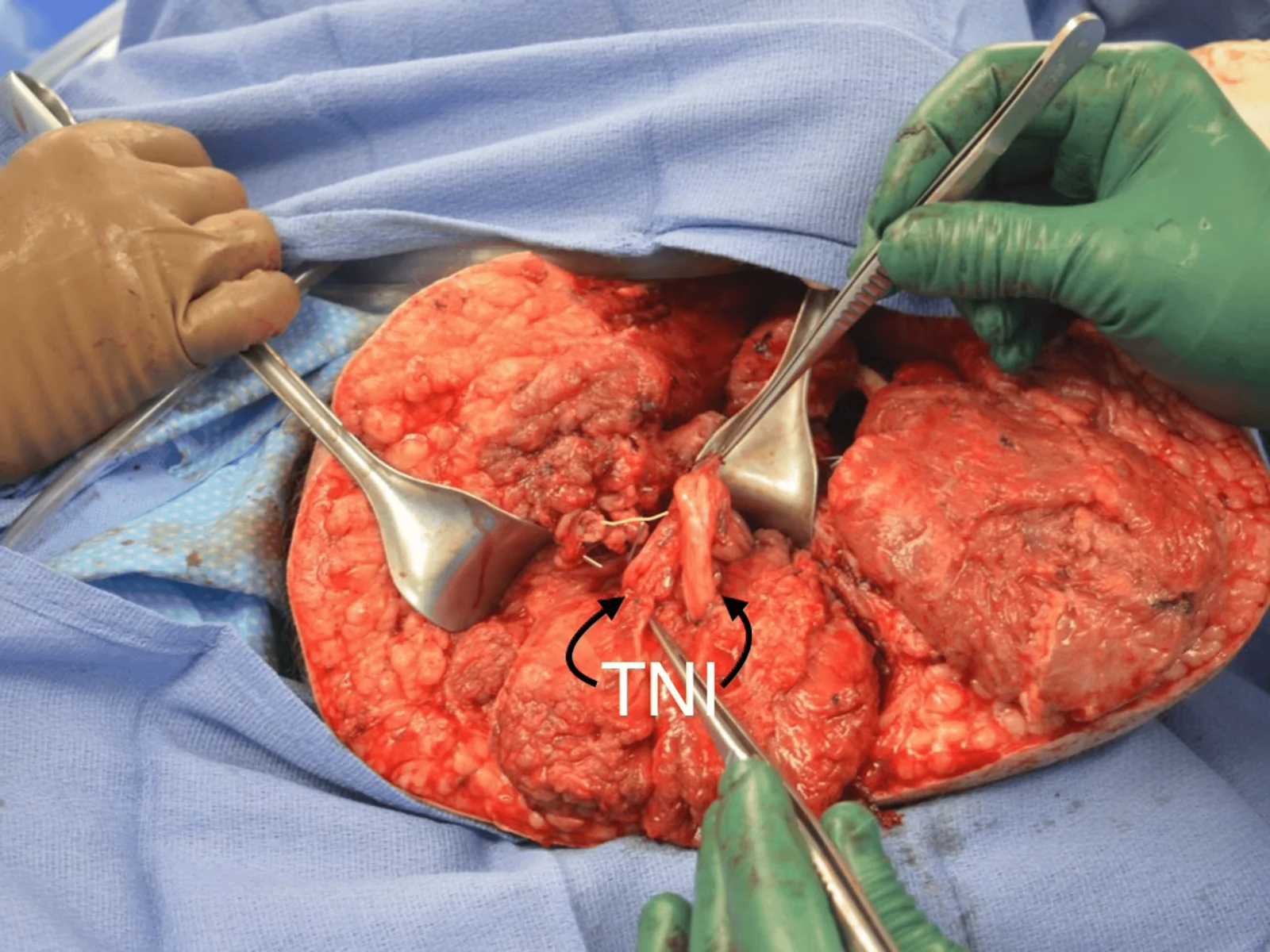
Intraoperative view demonstrating dissection of the sciatic nerve with identification of a completed targeted nerve implantation (TNI) between the sciatic nerve and the motor branch of the biceps femoris.

Patients are typically seen at approximately two weeks for wound evaluation, followed by visits at six weeks, three months, six months, and one year depending on the clinical situation. Early rehabilitation is primarily dictated by the associated amputation or reconstruction rather than the TNI itself, and patients are not generally immobilized specifically to protect the TNI unless required by another component of the reconstruction. Once the wound is healed, therapy can progress with range of motion, edema control, desensitization, scar management, prosthetic preparation/use when applicable, and gradual return to activity.

Pain outcomes are followed longitudinally rather than assessed at the first postoperative visit, with attention to residual-limb/neuroma pain, phantom pain when applicable, analgesic requirements, function/prosthetic tolerance, and patient satisfaction. There is currently no validated TNI-specific rehabilitation protocol, and this remains an area for further study.

## Discussion

Targeted muscle reinnervation (TMR) has been shown to reduce neuroma formation and phantom limb pain by providing regenerating axons with a physiologic target through coaptation to expendable motor nerve branches [9]. However, in clinical practice, particularly in above-knee amputations, surgeons frequently encounter size-caliber mismatch or a limited number of suitable motor nerve branches, which may restrict the ability to perform traditional nerve-to-nerve coaptation. 

When suitable motor targets are unavailable due to caliber mismatch, limited nerve length, unfavorable positioning, or prior surgical disruption, alternative strategies such as TNI and RPNI may be considered. Compared with RPNI, TNI may offer several advantages, including shorter operative time, avoidance of donor site morbidity associated with muscle graft harvest, and elimination of graft-related complications such as resorption or failure [15]. However, in some scenarios RPNI or TNI may be considered.

TNI is not intended to replace standard nerve-to-nerve TMR, which remains the preferred method when suitable motor targets are available. Rather, it serves as an adjunctive strategy in cases where conventional coaptation is limited by anatomical constraint. These scenarios may include absence of suitable expendable motor branches, extensive scar formation, prior operative disruption, or unfavorable nerve length and orientation. In such cases, TNI provides an alternative method of directing regenerating axons into a denervated muscular environment, with the goal of reducing symptomatic neuroma formation and neuropathic pain. Figure 5 demonstrates the decision-making algorithm for performing TMR vs TNI vs RPNI.

**Figure 5:**
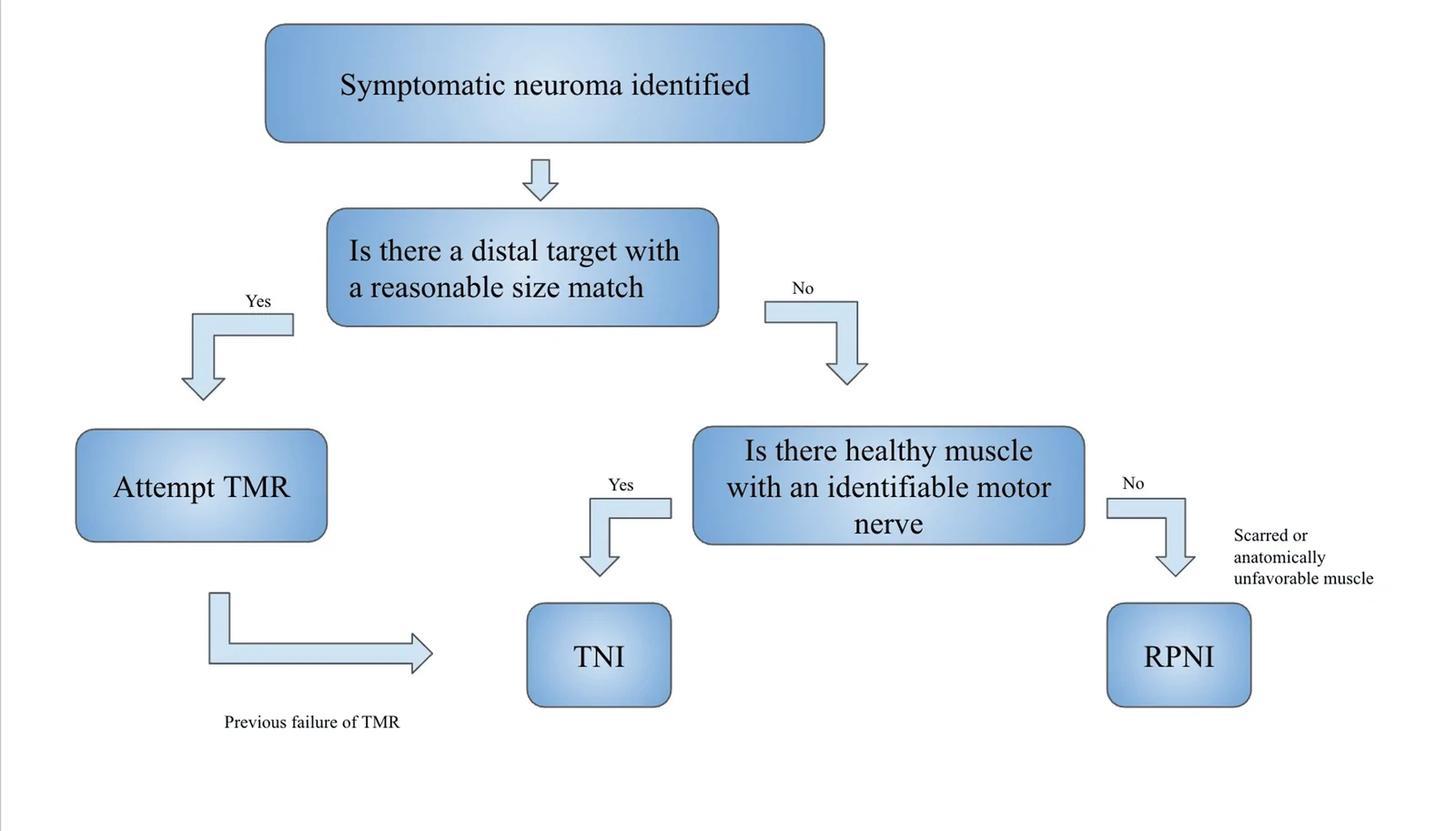
Image depicting the decision-tree for neuroma surgical interventions

As with other peripheral nerve procedures, TNI carries a range of potential complications that should be considered when counseling patients and interpreting outcomes. These include persistent neuropathic or residual-limb pain despite technically successful implantation, recurrent symptomatic neuroma, and incomplete improvement or recurrence of a focal Tinel sign. Technical complications specific to the implantation itself include nerve migration or pullout from the muscle target, and scar tethering or mechanical irritation of the implanted nerve. As with any dissection in this region, there is a risk of injury to adjacent motor nerves or unnecessary denervation of functional muscle, with potential resultant weakness if an inappropriate functional muscle or motor territory is sacrificed. Standard surgical complications including hematoma, seroma, infection, and wound dehiscence also apply, along with temporary postoperative pain or dysesthesia. TNI may fail to improve phantom limb pain, particularly when central mechanisms are a significant component, and some patients may require revision surgery or conversion to an alternative strategy such as TMR or RPNI. Many of these represent theoretical or generally recognized risks of peripheral nerve surgery rather than complications specifically observed in our series, and we would note this distinction in the text so as not to overstate the currently available TNI-specific complication literature.

This study is limited by lack of longitudinal clinical outcomes. Further investigation is warranted to evaluate postoperative pain control, neuroma formation, phantom limb pain, prosthetic fitment tolerance, psychological impact on treatment of PLP and neuroma pain, and patient reported outcomes following TNI. Future studies, including larger case series, should additionally assess demographic and operative variables such as amputation level, patient age, gender, timing of TNI relative to amputation, and prior limb salvage or revision procedures.

## Conclusions

Post-amputation neuropathic pain has a significant negative impact on psychological and physical morbidity. Pharmacological therapies have demonstrated minimal efficacy, whereas surgical techniques, including TNI, TMR, and RPNI, have shown promise for pain resolution. TMR is typically the first-line surgical approach for post-amputation neuromas, though patient factors may make this technique unsuitable. In cases where the anatomy is unfavorable for TMR, RPNI or TNI may be considered, and TNI, in particular, may offer shorter operative times and eliminate graft-related complications.
